# Groupyr: Sparse Group Lasso in Python

**DOI:** 10.21105/joss.03024

**Published:** 2021-02-24

**Authors:** Adam Richie-Halford, Manjari Narayan, Noah Simon, Jason Yeatman, Ariel Rokem

**Affiliations:** 1 eScience Institute, University of Washington; 2 Department of Psychiatry and Behavioral Sciences, Stanford University; 3 Department of Psychology, University of Washington; 4 Department of Biostatistics, University of Washington; 5 Graduate School of Education and Division of Developmental and Behavioral Pediatrics, Stanford University

## Abstract

For high-dimensional supervised learning, it is often beneficial to use domain-specific knowledge to improve the performance of statistical learning models. When the problem contains covariates which form groups, researchers can include this grouping information to find parsimonious representations of the relationship between covariates and targets. These groups may arise artificially, as from the polynomial expansion of a smaller feature space, or naturally, as from the anatomical grouping of different brain regions or the geographical grouping of different cities. When the number of features is large compared to the number of observations, one seeks a subset of the features which is sparse at both the group and global level.

The sparse group lasso (Simon et al., 2013) is a penalized regression technique designed for exactly these situations. It combines the original lasso (Tibshirani, 1996), which induces global sparsity, with the group lasso (Yuan & Lin, 2006), which induces group-level sparsity. It estimates a target variable $\hat{y}$ from a feature matrix **X**, using

$$\hat{y}=X\hat{\beta },$$

as depicted in Figure 1, with color encoding the group structure of the covariates in **X**. The coefficients in $\hat{\beta }$ characterize the relationship between the features and the target and must satisfy (Simon et al., 2013)

$$\hat{\beta }=\underset{\beta }{\text{min}}\frac{1}{2}{\left\|y-\overset{G}{\underset{\ell =1}{\sum }}X^{\left(\ell \right)}\beta^{\left(\ell \right)}\right\|}_{2}^{2}+(1-\lambda )\alpha \overset{G}{\underset{\ell =1}{\sum }}\sqrt{p_{\ell }}{\left\|\beta^{\left(\ell \right)}\right\|}_{2}+\lambda \alpha \|\beta {\|}_{1},$$

where *G* is the total number of groups, **X**^(*ℓ*)^ is the submatrix of **X** with columns belonging to group *ℓ*, *β*^(*ℓ*)^ is the coefficient vector of group *ℓ*, and *p*_*ℓ*_ is the length of *β*^(*ℓ*)^. The model hyperparameter *λ* controls the combination of the group-lasso and the lasso, with *λ* = 0 giving the group lasso fit and *λ* = 1 yielding the lasso fit. The hyperparameter *α* controls the overall strength of the regularization.

## Statement of need

*Groupyr* is a Python library that implements the sparse group lasso as scikit-learn (Buitinck et al., 2013; Pedregosa et al., 2011) compatible estimators. It satisfies the need for grouped penalized regression models that can be used interoperably in researcher’s real-world scikit-learn workflows. Some pre-existing Python libraries come close to satisfying this need. Lightning (Blondel & Pedregosa, 2016) is a Python library for large-scale linear classification and regression. It supports many solvers with a combination of the L1 and L2 penalties. However, it does not allow the user to specify groups of covariates (see, for example, this GitHub issue). Another Python package, group_lasso (Moe, 2020), is a well-designed and well-documented implementation of the sparse group lasso. It meets the basic API requirements of scikit-learn compatible estimators. However, we found that our implementation in *groupyr*, which relies on the *copt* optimization library (Fabian Pedregosa, 2020), was between two and ten times faster for the problem sizes that we encounter in our research (see the repository’s examples directory for a performance comparison). Additionally, we needed estimators with built-in cross-validation support using both grid search and sequential model based optimization strategies. For example, the speed and cross-validation enhancements were crucial to using *groupyr* in *AFQ-Insight*, a neuroinformatics research library (Richie-Halford et al., 2019).

## Usage

*Groupyr* is available on the Python Package Index (PyPI) and can be installed with


pip install groupyr


*Groupyr* is compatible with the scikit-learn API and its estimators offer the same instantiate, fit, predict workflow that will be familiar to scikit-learn users. See the online documentation for a detailed description of the API and examples in both classification and regression settings. Here, we describe only the key differences necessary for scikit-learn users to get started with *groupyr*.

For syntactic parallelism with the scikit-learn ElasticNet estimator, we use the keyword l1_ratio to refer to SGL’s *λ* hyperparameter. In addition to keyword parameters shared with scikit-learn’s ElasticNet, ElasticNetCV, LogisticRegression, and LogisticRe gressionCV estimators, users must specify the group assignments for the columns of the feature matrix X. This is done during estimator instantiation using the groups parameter, which accepts a list of numpy arrays, where the *i*-th array specifies the feature indices of the *i*-th group. If no grouping information is provided, the default behavior assigns all features to one group.

*Groupyr* also offers cross-validation estimators that automatically select the best values of the hyperparameters *α* and *λ* using either an exhaustive grid search (with tuning_strateg y=“grid”) or sequential model based optimization (SMBO) using the scikit-optimize library (with tuning_strategy=“bayes”). For the grid search strategy, our implementation is more efficient than using the base estimator with scikit-learn’s GridSearchCV because it makes use of warm-starting, where the model is fit along a pre-defined regularization path and the solution from the previous fit is used as the initial guess for the current hyperparameter value. The randomness associated with SMBO complicates the use of a warm start strategy; it can be difficult to determine which of the previously attempted hyperparameter combinations should provide the initial guess for the current evaluation. However, even without warm-starting, we find that the SMBO strategy usually outperforms grid search because far fewer evaluations are needed to arrive at the optimal hyperparameters. We provide examples of both strategies (grid search for a classification example and SMBO for a regression example) in the online documentation.

## Figures and Tables

**Figure 1:**
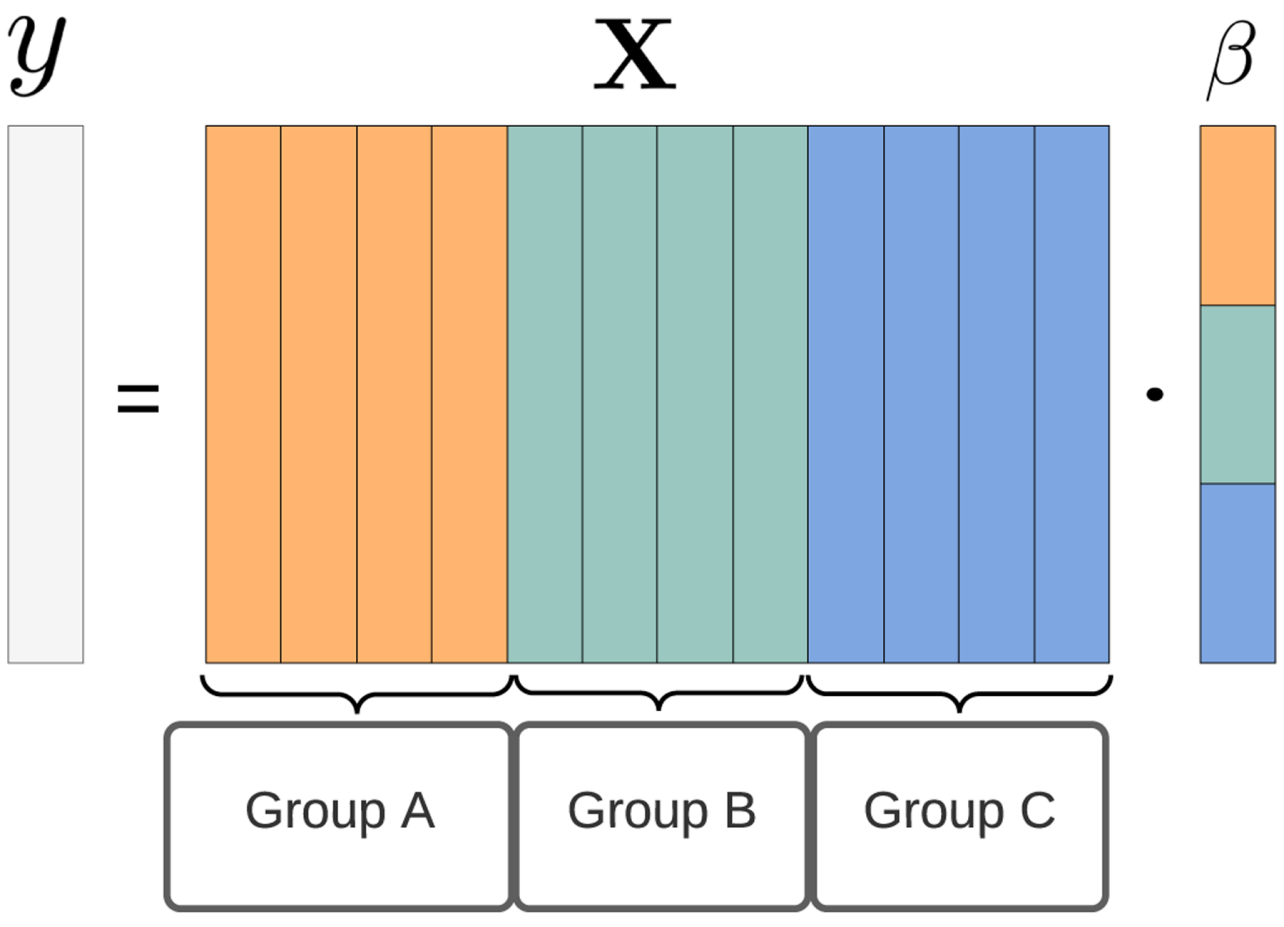
A linear model, *y* = **X** · *β*, with grouped covariates. The feature matrix **X** is color-coded to reveal a group structure. The coefficients in *β* follow the same grouping.

## References

[R1] Blondel M, & Pedregosa F (2016). Lightning: large-scale linear classification, regression and ranking in Python. 10.5281/zenodo.200504

[R2] Brand A, Allen L, Altman M, Hlava M, & Scott J (2015). Beyond authorship: Attribution, contribution, collaboration, and credit. Learned Publishing, 28(2), 151–155. 10.1087/20150211

[R3] Buitinck L, Louppe G, Blondel M, Pedregosa F, Mueller A, Grisel O, Niculae V, Prettenhofer P, Gramfort A, Grobler J, Layton R, VanderPlas J, Joly A, Holt B, & Varoquaux G (2013). API design for machine learning software: Experiences from the scikit-learn project. ECML PKDD Workshop: Languages for Data Mining and Machine Learning, 108–122. http://arxiv.org/abs/1309.0238

[R4] Fabian Pedregosa GD, Geoffrey Negiar. (2020). Copt: Composite optimization in python. 10.5281/zenodo.1283339

[R5] Moe YM (2020). Group lasso (Version swh:1:dir:18ab9abeda24c3466411280c15c740ab1cbe2f00). https://github.com/yngvem/group-lasso

[R6] Pedregosa F, Varoquaux G, Gramfort A, Michel V, Thirion B, Grisel O, Blondel M, Prettenhofer P, Weiss R, Dubourg V, Vanderplas J, Passos A, Cournapeau D, Brucher M, Perrot M, & Duchesnay É (2011). Scikit-learn: Machine learning in Python. Journal of Machine Learning Research, 12, 2825–2830.

[R7] Richie-Halford A, Yeatman J, Simon N, & Rokem A (2019). Multidimensional analysis and detection of informative features in diffusion MRI measurements of human white matter. bioRxiv. 10.1101/2019.12.19.882928 PMC8270416 34181648

[R8] Simon N, Friedman J, Hastie T, & Tibshirani R (2013). A sparse-group lasso. Journal of Computational and Graphical Statistics, 22(2), 231–245. 10.1080/10618600.2012.681250

[R9] Tibshirani R (1996). Regression Shrinkage and Selection Via the Lasso. Journal of the Royal Statistical Society: Series B (Methodological), 58(1), 267–288. 10.1111/j.2517-6161.1996.tb02080.x

[R10] Yuan M, & Lin Y (2006). Model selection and estimation in regression with grouped variables. Journal of the Royal Statistical Society: Series B (Statistical Methodology), 68(1), 49–67. 10.1111/j.1467-9868.2005.00532.x

